# Effect of cold agglutinins on red blood cell parameters in a trauma patient: a case report

**DOI:** 10.11613/BM.2018.031001

**Published:** 2018-10-15

**Authors:** Anita Topic, Lara Milevoj Kopcinovic, Ana Bronic, Marina Pavic

**Affiliations:** Department of clinical chemistry, Sestre milosrdnice University Hospital Center, Zagreb, Croatia

**Keywords:** cold agglutinins, preanalytical errors, red blood cell count, spurious complete blood count results

## Abstract

The presence of cold agglutinins (CAs) in samples intended for complete blood count (CBC) using automated haematology analysers might cause serious preanalytical errors. In this report we describe the case of a 90-year old female patient admitted to the Emergency department following trauma injuries. A blood testing on admission revealed surprisingly low red blood cell count (0.99 x 10^12^/L), low haematocrit (0.102 L/L) which did not correlate with haemoglobin concentration (100 g/L), and high erythrocytes indices (mean corpuscular haemoglobin, 101 pg; mean corpuscular haemoglobin concentration, 980 g/L). In the second sample, after repeated collection, almost equal results were observed. Blood smear examination under the microscope revealed clusters of erythrocytes. Cold agglutinins presence was suspected and, in order to get valid results, sample was warmed to 37 °C. Correction of CBC was observed. Furthermore, we performed some additional analysis to confirm the presence of CAs in this patient. The aim of this report was to present the laboratory findings in a case of CAs and propose a laboratory procedure for whole blood samples with suspected CAs.

## Introduction

The advent of automated haematology analysers has resulted in high throughput and improved accuracy of complete blood count (CBC) results. However, various pre-analytical and analytical factors, as well as clinical conditions might affect the reliability of CBC results, including white blood cell count (WBC), red blood cell count (RBC) and RBC indices (MCV; mean corpuscular volume, MCH; mean corpuscular haemoglobin, MCHC; mean corpuscular haemoglobin concentration), and platelet count (Plt).

Cold agglutinins (CAs) are the most commonly immunoglobulin (Ig) M antibodies (rarely IgA or IgG) directed against polysaccharide antigens located on the surface of erythrocytes which become activated by low temperature. The resulting antigen-antibody complex strongly activates the classical complement cascade, which might cause removal of RBCs in the reticuloendothelial system, intravascular and extravascular haemolysis (*1*). Cold agglutinin disease (CAD) is a type of autoimmune haemolytic anaemia (AIHA) characterized by an immune reaction against RBC self-antigens. A cold exposure or a concurrent infection may be sufficient to trigger the clinical manifestation of disease (*2*).

Cold agglutinins presence in samples intended for CBC analysis using automated haematology analysers might cause serious preanalytical errors. Most of the published reports on CAs presence are presented from a clinical perspective. Laboratory findings are usually scarce and the analytical perspective is often omitted. Several cases reported the importance of preanalytical effect of CAs on laboratory results, especially on CBC (*3*-*5*). The aim of this report is to present the laboratory findings found in a case of CAs and propose a laboratory procedure for whole blood samples with suspected CAs.

## Case report

A 90-year old female patient was admitted to the Emergency department (ED) of our hospital in December 2017 after trauma injuries. Following initial examination and due to nature of injuries, she was transferred to our dislocated traumatology unit where further diagnostic examinations were performed.

### Laboratory analyses

As a part of diagnostic processing, samples were referred to our laboratory for routine haematology, coagulation, biochemistry, blood gas and urine analysis.

Peripheral blood for CBC, collected by venipuncture in 3.5 mL Vacuette^®^ tube (K_3_EDTA, Greiner Bio-One, Kremsmunster, Austria), was analysed on Sysmex XT-1800i haematology analyser (Sysmex Corporation, Kobe, Japan). The results on admission are presented in Table 1, Sample 1. Red blood cell count and haematocrit (Hct) values were surprisingly low and did not correlate with the haemoglobin (Hb) concentration. Consequently, RBC indices were spuriously increased, especially MCH and MCHC, while Plt and WBC count seemed valid. Additionally, the RBC results were flagged by the analyser. Because of flags which indicated RBC agglutination and the obviously erroneous RBC results, the tube was visually checked for clumps and the presence of micro-aggregates was established. After centrifugation, haemolysis of the sample was noted. According to our laboratory protocol, these results were not released to the clinician and a new sample was requested from the ward.

**Table 1:** Laboratory values of haematology parameters at patient admission to Traumatology clinic

**Parameter**	Sample 1*	**Sample 2^†^**	**Sample 2a^‡^**	**Sample 2b^§^**	**Reference interval**
RBC (x10^12^/L)	0.99	1.73	2.79	2.98	3.86 - 5.08
Hb (g/L)	100	77	84	90	119 - 157
Hct(L/L)	0.102	0.169	0.252	0.277	0.356 - 0.470
MCV (fL)	103.0	97.7	90.3	93.0	83.0 - 97.2
MCH (pg)	101.0	44.5	30.1	30.2	27.4 - 33.9
MCHC (g/L)	980	456	333	325	320 - 345
RDW (%)	23.9	18.2	17.2	17.2	9.0 - 15.0
Plt (x10^9^/L)	278	206	258	272	158 - 424
MPV (fL)	10.3	9.1	9.8	10.2	6.8 - 10.4
WBC (x10^9^/L)	6.0	5.3	6.7	7.1	3.4 - 9.7
*First sample after admission. ^†^Second (repeated collection) sample. ^‡^Second sample incubated at 37 ºC for 30 minutes. ^§^Second sample with Cellpack^®^ replaced plasma. RBC - red blood cells. Hb - haemoglobin. Hct - haematocrit. MCV - mean corpuscular volume. MCH - mean corpuscular haemoglobin. MCHC - mean corpuscular haemoglobin concentration. RDW - red cell distribution width. Plt - platelets. MPV - mean platelet volume. WBC - white blood cells.					

**Figure 1:**
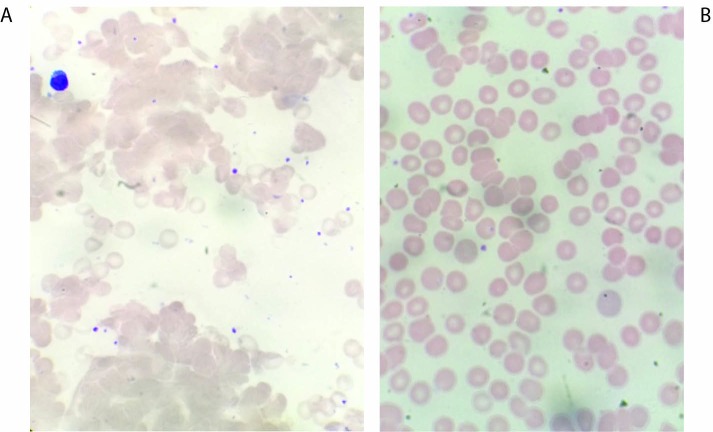
Peripheral blood smear, May-Grünwald-Giemsa stain, 1000x. A: Sample after repeated collection presenting with erythrocyte clusters. B: Sample after incubation at 37 °C for 30 minutes without showing erythrocyte clusters.

A new whole blood sample was delivered to our laboratory. After visual inspection of the sample for clumps, we excluded their presence and CBC testing was repeated. The test results were almost equal to those measured in the first sample (Table 1, Sample 2). Again, low RBC and Htc with high MCH and MCHC values were observed. The analyser’s flags were the same. Blood smear was prepared, using the May-Grünwald-Giemsa stain (Merck, Darmstadt, Germany), and examination under the light microscope (Olympus BX43, Olympus, Tokyo, Japan) revealed clusters of RBCs (Figure 1A). At this point, the presence of CAs was suspected and the tube was incubated at 37 °C for 30 minutes. The CBC results after incubation seemed corrected (Table 1, Sample 2a). Clusters of RBCs were not observed in the blood smear prepared from the warmed sample (Figure 1B). According to our intralaboratory procedure based on Sysmex’s document for flagging interpretation, we centrifuged the warmed sample for 10 min at 1800xg and replaced the supernatant plasma with the same volume of particle-free commercial diluent (Cellpack^®^, Sysmex Corporation, Kobe, Japan) (*6*). This procedure is sometimes required in cases with high CAs titers. The sample was well mixed and CBC test was run again. The results obtained were comparable to those obtained by incubating the sample at 37 °C (Table 1, Sample 2b).

### Further investigations

To confirm the presence of CAs, we performed some additional analyses. Direct and indirect antiglobulin (Coombs) test using anti-IgG and anti-C3 antibodies were performed using MONOGnost^®^ monoclonal polyspecific anti-human globulin reagent (Biognost, Zagreb, Croatia) and the results were positive for direct and negative for indirect antiglobulin test. The results indicated *in vivo* sensitization of patient RBCs with IgG and/or C3 component of complement. Furthermore, the concentrations of IgG, IgA, IgM, C3 and C4 component of complement in patient’s serum were determined on the Abbott Architect c8000 automated biochemistry analyser (Abbott Laboratories, IL, USA). Only concentration of IgM was above the upper reference limit which indicated that CAs in this case might be IgM class, such as usually found in CAD (Table 2).

**Table 2:** The patient’s laboratory results for immunoglobulins and component of complement concentrations

**Parameter**		**Reference interval**
**Immunoglobulin G, g/L**	13.70	5.52 - 16.31
**Immunoglobulin A, g/L**	1.71	0.69 - 5.17
**Immunoglobulin M, g/L**	4.26	0.33 - 2.93
**C3 component of complement, g/L**	1.07	0.83 - 1.93
**C4 component of complement, g/L**	0.24	0.15 - 0.57

To prevent future analytical problems related to the presence of CAs in the patient’s sample, we informed the clinical staff on the importance of immediate analysis of this patient’s sample (*i.e*. immediate sample transport after collection was instituted for this sample alone). The results of haematology parameters determined during the rest of the patient’s hospitalization period are presented in Table 3. There were no further analytical problems when the sample was immediately transported to the laboratory. On the 3rd day of hospitalization, to correct for anaemia, the patient received two doses of RBC concentrate.

**Table 3:** Laboratory values of haematology parameters during the rest of the patient’s hospitalization period

**Parameter**	**2nd day**	3rd day*	**4th day**	**5th day**	**6th day**
**RBC (x10^12^/L)**	2.76	3.44	3.44	3.37	3.96
**Hb (g/L)**	89	111	112	101	117
**Hct (L/L)**	0.267	0.321	0.321	0.309	0.361
**MCV (fL)**	96.7	93.3	93.3	91.7	91.2
**MCH (pg)**	32.2	32.3	32.6	30.0	29.5
**MCHC (g/L)**	333	346	349	327	324
**RDW (%)**	18.8	17.7	17.1	16.7	16.5
**Plt (x10^9^/L)**	273	237	244	249	284
**MPV (fL)**	9.3	9.9	9.3	9.8	9.5
**WBC (x10^9^/L)**	5.1	8.2	4.7	3.8	5.1
*Patient received two doses of RBC concentrate. All samples were immediately transported to the laboratory after collection. RBC - red blood cells. Hb - haemoglobin. Hct - haematocrit. MCV - mean corpuscular volume. MCH - mean corpuscular haemoglobin. MCHC - mean corpuscular haemoglobin concentration. RDW - red cell distribution width. Plt - platelet. MPV - mean platelet volume. WBC - white blood cells.					

## What happened?

Erroneous values of RBC count and RBC-dependent parameters in this case were the consequence of CAs presence in the patient’s sample. Cold agglutinins led to RBC micro-aggregates formation, which resulted in inaccurately low RBC count. Consequently, errors were also detectable on Htc and RBC indices values. Interestingly, we later found out that the CBC was also performed on admission to the ED where unusual results were not observed. In fact, RBC was 3.3x10^12^/L, Hb 98 g/L, Htc 0.309 L/L, MCV 93.1 fL, MCH 29.5 pg, MCHC 317 g/L. Since this case occurred in the winter period, it is possible that low temperature during the transport from one location to other triggered activation of CAs in this patient.

### Solution

To solve preanalytical interference of CAs and get valid results, we warmed the sample at body temperature in order to achieve separation of agglutinated RBCs. To prevent future analytical problems, samples of this patient were delivered to the laboratory immediately after collection and promptly analysed. Considering organisation at our location, we can ensure the delivery of sample within 5 minutes of collection so the usual transport procedure at body temperature in water bath has not been applied.

**Figure 2:**
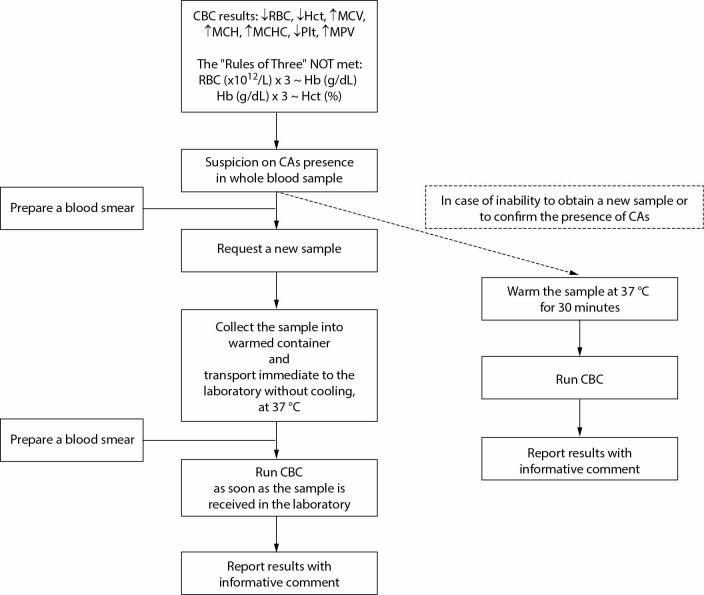
A proposed laboratory procedure for whole blood samples with suspected cold agglutinins. CBC - complete blood count. Hct - haematocrit. MCV - mean corpuscular volume. MCH - mean corpuscular haemoglobin. MCHC - mean corpuscular haemoglobin concentration. RDW - red cell distribution width. Plt - platelet. MPV - mean platelet volume. WBC - white blood cells. CAs - cold agglutinins.

A proposed laboratory procedure for whole blood samples with suspected CAs is presented in Figure 2. Spuriously low RBC, Hct and elevated MCV, MCH and, especially MCHC, as well as discrepancy between Hct, Hb and RBC results should always trigger suspicion of CAs presence in blood samples. One of the most indicative parameters generated by haematology analysers is MCHC, because of its mode of calculation. Its abnormalities alarm the user that the sample has to be checked. A quick visual check of the Hb, Hct and RBC results can be done by applying the “Rules of Three”. The rules say that the value of Hb should be aproximately three times the value of the RBC (RBC (10^12^/L) x 3 ~ Hb (g/dL)) and the value of Hct should be aproximately three times the value of the Hb (Hb (g/dL) x 3 ~ Hct (%)) (*7*). Although these rules apply only to specimens that have normocytic normochromic erythrocytes, they present a very simple and easy tool to detect possible samples with CAs within the large amount of routine samples. Effects of CAs are commonly visible on RBC count and RBC-dependent parameters, but it is important to stress that effects can be also visible on Plt causing spuriously low Plt count and falsely increased mean platelet volume (MPV). Therefore, such results should also raise the suspicion on CAs presence in the blood sample. To confirm RBC or Plt agglutination in the sample, the best way is blood smear examination under the microscope. As shown in Figure 2, the suspected presence of CAs has been raised, and the next step involves requesting a new sample and transporting that sample immediately after collection to the laboratory. The tube for blood collection should be warmed and transported to the laboratory, without cooling, at 37 °C, in an insulated container filled with warmed water and in a styrofoam box. The analysis should be made as soon as the sample is received in the laboratory. After the presence of CAs is evidenced, on the test result report informative comment should be incorporated. The comment should contain information to the clinician about finding CAs in the patient sample and information how to draw every next sample from that patient. In case of inability to obtain a new sample, warming the suspicious sample may be an alternative, but such results should be carefully interpreted, because it is not possible to be sure that all RBCs are separated after sample warming. In case of reporting results informative comment should also contain information that results were obtained after warming the sample. Also, warming the suspicious sample presents an easy evidence way of the presence of CAs.

## Discussion

In the presence of CAs, we found erroneously low RBC values and discrepancy between Hb and Hct. The analyser directly measures RBC, Htc and Hb parameters, while MCV, MCH and MCHC are calculated from measured parameters. Low RBC count was the result of formed RBCs microaggregates in the sample. These microaggregates the analyser may count as WBC or single RBC while, very large aggregates may exclude from the count. All of these lead to an erroneously low RBC count and, consequently, abnormal results of other CBC parameters (haematocrit, MCV, MCH and MCHC) (*3*). The analyser calculates MCH and MCHC indices using three measured parameters. Such, MCH is calculated as a quotient of haematocrit and RBC count (MCH, pg = Hct/RBC), while MCHC is calculated as a quotient of haemoglobin and haematocrit (MCHC, g/L = Hb/Hct). MCHC might provide a mean for quality control of these parameters. High MCHC is a good indicator of analysis or sample error (haemolysis, lipaemia, agglutination of erythrocytes, *etc.*). Contemporary automated haematology analysers display flags to warn for possible errors. In our case, MCHC value was extremely high and flags from the analyser indicated RBC agglutination. Agglutination, usually, can be visually observed, but the blood smear examination is the best way to confirm it. The erroneous results were corrected after the sample was warmed to 37 ºC. Warming the sample leads to elution of IgM antibody from the cell surface allowing the agglutinated RBCs to separate. In a published report by Nikousefat *et al*., a female patient with low RBC count, incompatible Hct and Hb, and increased RBC indices measured on automated Sysmex analyser, was diagnosed with CAD and warming the sample to 37 ºC led to correct results (*8*). Similar results were found in a report described by Ercan S. *et al.* using the ABX Pentra 80 haematology analyser (*4*). They evaluated the CAs antibody titre and valid laboratory results were obtained after proper sample transport in warmed container and immediate analysis on haematology analyser. Erroneously low RBC count, low Htc that did not correlate with the Hb concentration and increased RBC indices in the presence of CAs were also described by Kakkar, using Advia 60 haematology analyser, and Yasar and Breuer *et al*., using automated Coulter haematology analysers (*3*, *5*, *9*).

White blood cell and Hb concentrations were unaffected by CAs. Haemoglobin is measured directly by the lysis of RBCs in another channel. Concentration can be falsely increased in haemolytic samples (Sample 1, Table 1). Platelet count and indices can be measured incorrectly because Plt can also undergo auto-agglutination. Such case is reported by Yasar *et al*. who described unmeasurable Plt and MPV, but after warming the sample to 37 ºC the parameters were normally measured (*3*).

Diagnosis of CAD requires further analysis. We performed some of them; direct and indirect antiglobulin (Coombs) test, serum concentrations of immunoglobulins and C3 and C4 components of complement. We found positive direct and negative indirect antiglobulin test and high concentration of IgM. Positive Coombs test indicated that erythrocytes are attached with IgG and/or C3 component of complement. In the study on 58 patients, positivity for anti-C3d antibodies was 74%, anti-C3d + anti-IgG 22% and anti-IgG 3.4% (*10*). Except the problems with routine laboratory analysis, CAs causes difficulty in blood type detecting. Lodi *et al*. reported the case of 48-year-old patient whose blood group was not determined due to analytical problems causing by CAs presence in patient’s sample and the patient died after receiving emergency transfusion of universal RBCs (*11*). Cold agglutinins and CAD are rare, according to a Norwegian study the prevalence is 16 cases per million inhabitants and incidence rate is one *per* million *per* year. Most often affected are adults in the seventh decade of life with slight female predominance (*12*). Cold agglutinins are commonly IgM antibodies activated at low temperatures. Since IgM are the largest human antibodies their hexameric (or pentameric) forms have antigen-binding sites sufficiently far apart to bridge the distance imposed upon RBC in suspensions like plasma, thus allowing spontaneous agglutination. Cold agglutinins, found in low titres in healthy individuals, have no activity at temperatures higher than 4 ºC. Pathological CAs usually react at 28 to 31 ºC (*13*). Blood cell counts might also be affected at low temperature by cryoglobulins. Cryoglobulins are circulating immunoglobulins that become insoluble and precipitate in temperature between 4 °C and 37 °C causing analytical interference for automated blood cell counters, mainly resulting in pseudoleukocytosis and pseudothrombocytosis (*14*-*16*). Passing through the aperture of the analyser, cryoglobulin particles might be counted as WBCs or Plt when they approximate their size, structure, and shape. Unlike CAs, cryoglobulins usually slightly affect RBC count and Hb measurement (*14*). Therefore, CAs and cryoglobulins differentially interfere on blood cell counts, however laboratory procedure how to analyse such samples is the same in both cases.

## What YOU should / can do in your laboratory to prevent such errors

A proposed laboratory procedure to easily detect and prevent preanalytical errors caused by CAs presence in whole blood samples is presented in Figure 2.
